# 3,7-Dihydr­oxy-3,7-diphenyl-2*H*,6*H*-pyrrolo[3,4-*f*]isoindole-1,5(3*H*,7*H*)-dione methanol disolvate

**DOI:** 10.1107/S1600536808039445

**Published:** 2008-11-29

**Authors:** Shan Liu, Jing-Ning Liu, Peng Jiang, Qing-Yan Chu, Hong-Jun Zhu

**Affiliations:** a Department of Applied Chemistry, College of Science, Nanjing University of Technology, Nanjing 210009, People’s Republic of China; b Department of Public Security Science, Jiangsu Police Institute, Nanjing 210012, People’s Republic of China

## Abstract

The asymmetric unit of the title compound, C_22_H_16_N_2_O_4_·2CH_4_O, contains one half-mol­ecule and a methanol solvent mol­ecule. The aromatic ring is oriented at a dihedral angle of 82.91 (3)° with respect to the planar indole ring systems. In the crystal structure, inter­molecular O—H⋯O and N—H⋯O hydrogen bonds link the mol­ecules into chains along the *b* axis.

## Related literature

For general background, see: Antoniadis *et al.* (1994 ▶); Kolosov *et al.* (2002 ▶); Tonzola *et al.* (2003 ▶). For a related structure, see: Liu *et al.* (2008 ▶). For bond-length data, see: Allen *et al.* (1987 ▶).
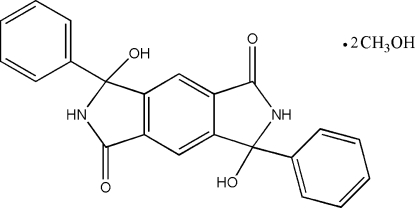


## Experimental

### 

#### Crystal data

C_22_H_16_N_2_O_4_·2CH_4_O
*M* *_r_* = 436.20
Monoclinic, 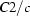

*a* = 17.767 (4) Å
*b* = 6.6300 (13) Å
*c* = 20.215 (4) Å
β = 106.59 (3)°
*V* = 2282.1 (9) Å^3^
*Z* = 4
Mo *K*α radiation
μ = 0.09 mm^−1^
*T* = 298 (2) K
0.30 × 0.20 × 0.10 mm

#### Data collection

Enraf–Nonius CAD-4 diffractometer
Absorption correction: ψ scan (North *et al.*, 1968 ▶) *T*_min_ = 0.973, *T*_max_ = 0.991
4477 measured reflections
2245 independent reflections
1050 reflections with *I* > 2σ(*I*)
*R*_int_ = 0.075
3 standard reflections frequency: 120 min intensity decay: none

#### Refinement

*R*[*F* ^2^ > 2σ(*F* ^2^)] = 0.070
*wR*(*F* ^2^) = 0.156
*S* = 1.04
2245 reflections
152 parameters
H atoms treated by a mixture of independent and constrained refinement
Δρ_max_ = 0.21 e Å^−3^
Δρ_min_ = −0.25 e Å^−3^

### 

Data collection: *CAD-4 Software* (Enraf–Nonius, 1985 ▶); cell refinement: *CAD-4 Software*; data reduction: *XCAD4* (Harms & Wocadlo, 1995 ▶); program(s) used to solve structure: *SHELXS97* (Sheldrick, 2008 ▶); program(s) used to refine structure: *SHELXL97* (Sheldrick, 2008 ▶); molecular graphics: *SHELXTL* (Sheldrick, 2008 ▶); software used to prepare material for publication: *SHELXTL*.

## Supplementary Material

Crystal structure: contains datablocks I, global. DOI: 10.1107/S1600536808039445/hk2579sup1.cif

Structure factors: contains datablocks I. DOI: 10.1107/S1600536808039445/hk2579Isup2.hkl

Additional supplementary materials:  crystallographic information; 3D view; checkCIF report

## Figures and Tables

**Table 1:** Hydrogen-bond geometry (Å, °)

*D*—H⋯*A*	*D*—H	H⋯*A*	*D*⋯*A*	*D*—H⋯*A*
O1—H1⋯O3	0.82	1.86	2.633 (4)	156
O3—H3⋯O2^i^	0.82	1.94	2.719 (4)	158
N—H⋯O1^ii^	0.84 (4)	2.08 (4)	2.907 (4)	170

Symmetry codes: (i) 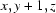; (ii) 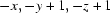.

**Fig. 1.**
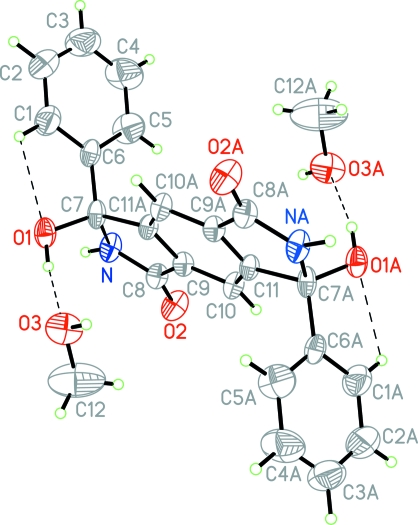
The molecular structure of the title molecule, with the atom-numbering scheme. Displacement ellipsoids are drawn at the 40% probability level [symmetry code: (A) 1/2 - x, 3/2 - y, 1 - z]. Hydrogen bonds are shown as dashed lines.

**Fig. 2.**
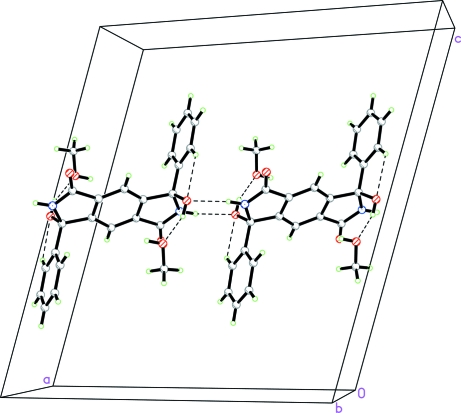
A partial packing diagram of the title compound. Hydrogen bonds are shown as dashed lines.

## supplementary crystallographic information

### Comment

The title compound is an important intermediate used to synthesize the monomer 2,5-dibenzoyl-1,4-phenylenediamine, which can be utilized to synthesize organic semiconductors and conjugated polymers (Tonzola *et al.*, 2003), which are of wide current interest for applications in electronic and optoelectronic devices including light-emitting diodes (Kolosov *et al.*,  2002), thin film transistors and photovoltaic cells (Antoniadis *et al.*,  1994). We report herein the crystal structure of the title compound, which is of interest to us in the field.

The asymmetric unit of the title compound (Fig. 1) contains one-half molecule and a methanol molecule, where the bond lengths (Allen *et al.*, 1987) and angles are within normal ranges. Rings A (C1-C6), B (N/C7-C8/C11A) and C (C9-C11/C9A-C11A) are, of course, planar, and the dihedral angle between rings B and C is B/C = 1.66 (3)° [symmetry code: (A) 1/2 - x, 3/2 - y, 1 - z]. So, the indole ring is essentially planar. Ring A is oriented with respect to the planar indole ring at a dihedral angle of 82.91 (3)°. The intramolecular C-H···O hydrogen bond (Table 1) results in the formation of a nonplanar five-membered ring D (O1/C1/C6/C7/H1B) adopting envelope conformation with O1 atom displaced by 0.288 (3) Å from the plane of the other ring atoms.

In the crystal structure, intermolecular O-H···O and N-H···O hydrogen bonds (Table 1) link the molecules into chains along b axis (Fig. 2), in which they may be effective in the stabilization of the structure.

### Experimental

The title compound was prepared according to the literature method (Liu *et al.*,  2008). Crystals suitable for X-ray analysis were obtained by dissolving the title compound (0.5 g) in methanol (50 ml), and evaporating the solvent slowly at room temperature for about 30 d.

### Refinement

H atom (for NH) was located in difference synthesis and refined isotropically [N-H = 0.84 (4) Å and U_iso_(H) = 0.042 (11) Å^2^]. Remaining H atoms were positioned geometrically, with O-H = 0.82 Å (for OH) and C-H = 0.93 and 0.96 Å for aromatic and methyl H, respectively, and constrained to ride on their parent atoms with U_iso_(H) = xU_eq_(C,O), where x = 1.2 for aromatic H and x = 1.5 for all other H atoms.

### Crystal data

**Table:**

C_22_H_16_N_2_O_4_·2CH_4_O	*F*_000_ = 920
*M**_r_* = 436.20	*D*_x_ = 1.270 Mg m^−^^3^
Monoclinic, *C*2/*c*	Mo *K*α radiation λ = 0.71073 Å
Hall symbol: -C 2yc	Cell parameters from 25 reflections
*a* = 17.767 (4) Å	θ = 9–12º
*b* = 6.6300 (13) Å	µ = 0.09 mm^−^^1^
*c* = 20.215 (4) Å	*T* = 298 (2) K
β = 106.59 (3)º	Block, colorless
*V* = 2282.1 (9) Å^3^	0.30 × 0.20 × 0.10 mm
*Z* = 4	

### Data collection

**Table:**

Enraf–Nonius CAD-4 diffractometer	*R*_int_ = 0.075
Radiation source: fine-focus sealed tube	θ_max_ = 26.0º
Monochromator: graphite	θ_min_ = 2.1º
*T* = 298(2) K	*h* = −21→21
ω/2θ scans	*k* = 0→8
Absorption correction: ψ scan(North *et al.*, 1968)	*l* = −24→24
*T*_min_ = 0.973, *T*_max_ = 0.991	3 standard reflections
4477 measured reflections	every 120 min
2245 independent reflections	intensity decay: none
1050 reflections with *I* > 2σ(*I*)	

### Refinement

**Table:**

Refinement on *F*^2^	Secondary atom site location: difference Fourier map
Least-squares matrix: full	Hydrogen site location: inferred from neighbouring sites
*R*[*F*^2^ > 2σ(*F*^2^)] = 0.071	H atoms treated by a mixture of independent and constrained refinement
*wR*(*F*^2^) = 0.156	*w* = 1/[σ^2^(*F*_o_^2^) + (0.0378*P*)^2^ + 1.8*P*] where *P* = (*F*_o_^2^ + 2*F*_c_^2^)/3
*S* = 1.04	(Δ/σ)_max_ < 0.001
2245 reflections	Δρ_max_ = 0.21 e Å^−^^3^
152 parameters	Δρ_min_ = −0.25 e Å^−^^3^
Primary atom site location: structure-invariant direct methods	Extinction correction: none

### Special details

**Table:**

Geometry. All e.s.d.'s (except the e.s.d. in the dihedral angle between two l.s. planes) are estimated using the full covariance matrix. The cell e.s.d.'s are taken into account individually in the estimation of e.s.d.'s in distances, angles and torsion angles; correlations between e.s.d.'s in cell parameters are only used when they are defined by crystal symmetry. An approximate (isotropic) treatment of cell e.s.d.'s is used for estimating e.s.d.'s involving l.s. planes.
Refinement. Refinement of *F*^2^ against ALL reflections. The weighted *R*-factor *wR* and goodness of fit *S* are based on *F*^2^, conventional *R*-factors *R* are based on *F*, with *F* set to zero for negative *F*^2^. The threshold expression of *F*^2^ > σ(*F*^2^) is used only for calculating *R*-factors(gt) *etc*. and is not relevant to the choice of reflections for refinement. *R*-factors based on *F*^2^ are statistically about twice as large as those based on *F*, and *R*- factors based on ALL data will be even larger.

### Fractional atomic coordinates and isotropic or equivalent isotropic displacement parameters (Å^2^)

**Table:**

	*x*	*y*	*z*	*U*_iso_*/*U*_eq_	
N	0.10301 (18)	0.4308 (5)	0.49495 (17)	0.0429 (13)	
H	0.060 (2)	0.372 (5)	0.4881 (17)	0.042 (11)*	
O1	0.04932 (13)	0.7469 (4)	0.51464 (13)	0.0451 (7)	
H1	0.0443	0.7801	0.4745	0.068*	
O2	0.15728 (14)	0.2655 (4)	0.41962 (14)	0.0537 (8)	
O3	0.05976 (17)	0.9420 (4)	0.40414 (15)	0.0657 (9)	
H3	0.0982	1.0163	0.4117	0.099*	
C1	0.0891 (3)	0.6807 (7)	0.6511 (2)	0.0689 (14)	
H1B	0.0549	0.7834	0.6302	0.083*	
C2	0.1022 (3)	0.6438 (10)	0.7205 (3)	0.0911 (18)	
H2A	0.0771	0.7224	0.7459	0.109*	
C3	0.1513 (3)	0.4939 (10)	0.7521 (3)	0.0879 (17)	
H3A	0.1597	0.4684	0.7989	0.106*	
C4	0.1878 (3)	0.3827 (8)	0.7149 (3)	0.0908 (17)	
H4A	0.2218	0.2802	0.7362	0.109*	
C5	0.1753 (3)	0.4194 (7)	0.6454 (2)	0.0721 (14)	
H5A	0.2009	0.3412	0.6204	0.086*	
C6	0.12522 (19)	0.5704 (6)	0.61275 (19)	0.0404 (10)	
C7	0.11403 (18)	0.6157 (5)	0.53721 (19)	0.0373 (9)	
C8	0.15509 (19)	0.4032 (6)	0.45934 (19)	0.0392 (9)	
C9	0.21057 (18)	0.5789 (5)	0.47727 (17)	0.0343 (9)	
C10	0.27381 (18)	0.6211 (5)	0.45295 (18)	0.0364 (9)	
H10A	0.2892	0.5363	0.4225	0.044*	
C11	0.31286 (17)	0.7995 (5)	0.47725 (18)	0.0325 (8)	
C12	0.0478 (4)	0.8537 (9)	0.3413 (3)	0.127 (3)	
H12A	0.0132	0.7406	0.3377	0.191*	
H12B	0.0971	0.8085	0.3361	0.191*	
H12C	0.0247	0.9497	0.3058	0.191*	

### Atomic displacement parameters (Å^2^)

**Table:**

	*U*^11^	*U*^22^	*U*^33^	*U*^12^	*U*^13^	*U*^23^
O1	0.0334 (13)	0.0397 (16)	0.0656 (19)	−0.0020 (12)	0.0198 (13)	0.0084 (14)
O2	0.0631 (17)	0.0341 (16)	0.0733 (19)	−0.0195 (14)	0.0342 (15)	−0.0183 (15)
O3	0.078 (2)	0.0487 (19)	0.067 (2)	−0.0248 (16)	0.0150 (17)	0.0023 (16)
N	0.037 (2)	0.0300 (19)	0.068 (3)	−0.0188 (15)	0.0243 (18)	−0.0108 (16)
C1	0.067 (3)	0.080 (4)	0.066 (3)	0.022 (3)	0.030 (3)	0.006 (3)
C2	0.088 (4)	0.128 (5)	0.065 (4)	0.025 (4)	0.034 (3)	−0.010 (4)
C3	0.092 (4)	0.116 (5)	0.060 (4)	−0.015 (4)	0.028 (3)	0.011 (4)
C4	0.118 (5)	0.072 (4)	0.075 (4)	0.022 (3)	0.017 (3)	0.029 (3)
C5	0.096 (4)	0.049 (3)	0.074 (3)	0.020 (3)	0.029 (3)	0.006 (3)
C6	0.0344 (19)	0.036 (2)	0.057 (3)	−0.0061 (18)	0.0232 (19)	0.001 (2)
C7	0.0263 (17)	0.0260 (19)	0.063 (3)	−0.0051 (16)	0.0189 (18)	−0.0024 (19)
C8	0.039 (2)	0.030 (2)	0.051 (2)	−0.0055 (18)	0.0179 (19)	0.001 (2)
C9	0.0321 (18)	0.027 (2)	0.044 (2)	−0.0042 (16)	0.0120 (17)	−0.0015 (18)
C10	0.0365 (19)	0.0251 (19)	0.053 (2)	−0.0032 (17)	0.0222 (17)	−0.0049 (18)
C11	0.0281 (18)	0.0277 (19)	0.045 (2)	−0.0066 (15)	0.0161 (16)	−0.0002 (17)
C12	0.223 (8)	0.071 (4)	0.077 (4)	−0.022 (5)	0.026 (5)	−0.020 (3)

### Geometric parameters (Å, °)

**Table:**

N—C8	1.337 (4)	C6—C7	1.512 (5)
N—C7	1.475 (4)	C7—O1	1.410 (4)
N—H	0.84 (4)	C7—C11^i^	1.518 (4)
O1—H1	0.8200	C8—O2	1.224 (4)
O3—H3	0.8200	C8—C9	1.502 (5)
C1—C6	1.353 (5)	C9—C11^i^	1.373 (4)
C1—C2	1.377 (6)	C9—C10	1.378 (4)
C1—H1B	0.9300	C10—C11	1.389 (4)
C2—C3	1.357 (7)	C10—H10A	0.9300
C2—H2A	0.9300	C11—C9^i^	1.373 (4)
C3—C4	1.344 (7)	C11—C7^i^	1.518 (4)
C3—H3A	0.9300	C12—O3	1.359 (5)
C4—C5	1.380 (6)	C12—H12A	0.9600
C4—H4A	0.9300	C12—H12B	0.9600
C5—C6	1.376 (5)	C12—H12C	0.9600
C5—H5A	0.9300		
			
C8—N—C7	115.3 (3)	O1—C7—C6	108.0 (3)
C8—N—H	126 (2)	N—C7—C6	112.2 (3)
C7—N—H	116 (2)	O1—C7—C11^i^	111.9 (3)
C7—O1—H1	109.5	N—C7—C11^i^	100.1 (3)
C12—O3—H3	109.5	C6—C7—C11^i^	113.3 (3)
C6—C1—C2	121.2 (5)	O2—C8—N	127.7 (3)
C6—C1—H1B	119.4	O2—C8—C9	126.5 (3)
C2—C1—H1B	119.4	N—C8—C9	105.7 (3)
C3—C2—C1	120.6 (5)	C11^i^—C9—C10	123.6 (3)
C3—C2—H2A	119.7	C11^i^—C9—C8	108.3 (3)
C1—C2—H2A	119.7	C10—C9—C8	128.1 (3)
C4—C3—C2	119.1 (5)	C9—C10—C11	115.0 (3)
C4—C3—H3A	120.5	C9—C10—H10A	122.5
C2—C3—H3A	120.5	C11—C10—H10A	122.5
C3—C4—C5	120.6 (5)	C9^i^—C11—C10	121.4 (3)
C3—C4—H4A	119.7	C9^i^—C11—C7^i^	110.6 (3)
C5—C4—H4A	119.7	C10—C11—C7^i^	128.0 (3)
C6—C5—C4	120.7 (4)	O3—C12—H12A	109.5
C6—C5—H5A	119.6	O3—C12—H12B	109.5
C4—C5—H5A	119.6	H12A—C12—H12B	109.5
C1—C6—C5	117.7 (4)	O3—C12—H12C	109.5
C1—C6—C7	121.6 (4)	H12A—C12—H12C	109.5
C5—C6—C7	120.6 (3)	H12B—C12—H12C	109.5
O1—C7—N	111.4 (3)		
			
C6—C1—C2—C3	−0.5 (8)	C5—C6—C7—N	45.5 (4)
C1—C2—C3—C4	0.6 (9)	C1—C6—C7—C11^i^	110.5 (4)
C2—C3—C4—C5	−0.4 (9)	C5—C6—C7—C11^i^	−66.9 (4)
C3—C4—C5—C6	0.0 (8)	C7—N—C8—O2	−178.1 (4)
C2—C1—C6—C5	0.2 (7)	C7—N—C8—C9	1.1 (4)
C2—C1—C6—C7	−177.3 (4)	O2—C8—C9—C11^i^	179.1 (4)
C4—C5—C6—C1	0.1 (7)	N—C8—C9—C11^i^	−0.1 (4)
C4—C5—C6—C7	177.6 (4)	O2—C8—C9—C10	0.3 (6)
C8—N—C7—O1	116.9 (3)	N—C8—C9—C10	−178.9 (4)
C8—N—C7—C6	−122.0 (3)	C11^i^—C9—C10—C11	−0.7 (6)
C8—N—C7—C11^i^	−1.6 (4)	C8—C9—C10—C11	177.9 (3)
C1—C6—C7—O1	−14.0 (5)	C9—C10—C11—C9^i^	0.7 (6)
C5—C6—C7—O1	168.6 (3)	C9—C10—C11—C7^i^	177.4 (3)
C1—C6—C7—N	−137.1 (4)		

Symmetry codes: (i) −*x*+1/2, −*y*+3/2, −*z*+1.

### Hydrogen-bond geometry (Å, °)

**Table:**

*D*—H···*A*	*D*—H	H···*A*	*D*···*A*	*D*—H···*A*
O1—H1···O3	0.82	1.86	2.633 (4)	156
O3—H3···O2^ii^	0.82	1.94	2.719 (4)	158
N—H···O1^iii^	0.84 (4)	2.08 (4)	2.907 (4)	170
C1—H1B···O1	0.93	2.32	2.681 (5)	102

Symmetry codes: (ii) *x*, *y*+1, *z*; (iii) −*x*, −*y*+1, −*z*+1.
